# Succinate Dehydrogenase Upregulation Destabilize Complex I and Limits the Lifespan of *gas-1* Mutant

**DOI:** 10.1371/journal.pone.0059493

**Published:** 2013-03-28

**Authors:** Claire Pujol, Ivana Bratic-Hench, Marija Sumakovic, Jürgen Hench, Arnaud Mourier, Linda Baumann, Victor Pavlenko, Aleksandra Trifunovic

**Affiliations:** 1 Cologne Excellence Cluster on Cellular Stress Responses in Aging-Associated Diseases (CECAD), Cologne University, Cologne, Germany; 2 Department of Laboratory Medicine, Karolinska Institutet, Stockholm, Sweden; 3 Department of Neuropathology, Institute of Pathology, University Hospital Basel, Basel, Switzerland; 4 Max Planck Institute for Biology of Aging, Cologne, Germany; 5 Center for Molecular Medicine Cologne, Cologne, Germany; UMASS-Amherst/Tufts University School of Medicine, United States of America

## Abstract

Many *Caenorhabditis elegans* mutants with dysfunctional mitochondrial electron transport chain are surprisingly long lived. Both short-lived (*gas-1(fc21)*) and long-lived (*nuo-6(qm200)*) mutants of mitochondrial complex I have been identified. However, it is not clear what are the pathways determining the difference in longevity. We show that even in a short-lived *gas-1(fc21)* mutant, many longevity assurance pathways, shown to be important for lifespan prolongation in long-lived mutants, are active. Beside similar dependence on alternative metabolic pathways, short-lived *gas-1(fc21)* mutants and long-lived *nuo-6(qm200)* mutants also activate hypoxia-inducible factor –1α (HIF-1α) stress pathway and mitochondrial unfolded protein response (UPR^mt^). The major difference that we detected between mutants of different longevity, is in the massive loss of complex I accompanied by upregulation of complex II levels, only in short-lived, *gas-1(fc21)* mutant. We show that high levels of complex II negatively regulate longevity in *gas-1(fc21)* mutant by decreasing the stability of complex I. Furthermore, our results demonstrate that increase in complex I stability, improves mitochondrial function and decreases mitochondrial stress, putting it inside a “window” of mitochondrial dysfunction that allows lifespan prolongation.

## Introduction

Mitochondria are organelles found in almost every eukaryotic cell. They produce a bulk of cellular energy in the form of ATP, which is required for numerous processes in the cell. Majority of ATP is formed during oxidative phosphorylation (OXPHOS) through the mitochondrial respiratory chain (MRC), a series of large enzyme complexes that couples the transfer of electrons to the creation of a proton gradient across the inner mitochondrial membrane. The MRC system consists of five multi-subunit complexes that are embedded in the inner mitochondrial membrane. It is not surprising that mutations in the MRC subunits lead to a variety of disorders. Complex I (NADH ubiquinone oxidoreductase, CO I) deficiency is the most common cause of mitochondrial diseases in both children and adults. Mutations in CO I subunits encoded by either mitochondrial (mtDNA) or nuclear DNA cause infantile encephalomyopathies or multisystem disorders in adults. Clinical symptoms in these patients may vary from mild to fatal [1].

Mitochondrial dysfunction has been modeled in a number of different animal species. Defective OXPHOS often results in severe phenotypes or premature death in majority of animal models [2], [3]. However, studies in *C. elegans* showed that dysfunction in the mitochondrial respiratory chain is not necessarily lethal. Oddly enough, several *C. elegans* mutants, defective in either MRC functions or in transport of the MRC substrates, are long lived [4], [5], [6]. This class of long-lived mutants is named Mit mutants and they usually carry a loss-of-function or a reduced-in-function alteration in components of the canonical MRC. Most of them exhibit a 20–40% increase in the mean adult life span, but in some cases life extension can be on the order of 300% [4], [5], [6]. Studies on worms with decreased expression of different MRC components obtained after the RNAi treatment, showed that a complete ablation of major MRC subunits leads to an embryonic arrest or severe phenotype with sterility and shortened lifespan, and only moderate decrease of these proteins has a positive effect on the lifespan [7]. It was shown that within certain “window”, a decrease in the amount of single MRC subunit will cause lifespan prolongation, while any further reduction will result in the lifespan shortening [7]. In agreement, a mild decrease in the level of protein in most cases does not have an effect on the lifespan [7].

There is no obvious connection between a life-span extension and disruption of any particular MRC complex in worms. Exception is Complex II (succinate:ubiquinone reductase or succinate dehydrogenase, CO II) that consists of four nuclear encoded subunits whose deficiency always leads to lifespan shortening [8]. Possible explanation could be that CO II not only works as the MRC complex that channels electrons coming from the oxidation of succinate, to ubiquinone; but also serves as the succinate dehydrogenase of the tricarboxylic acid cycle (TCA). This later role is likely essential for the *C. elegans* survival, as a majority of metabolic processes in the cell coincide in the TCA cycle [8].

Although partial deficiency in different CO I subunits leads to an increase in *C. elegans* lifespan, worms with a mutation in *gas-1* live shorter and have decreased fecundity [9]. Mammalian homolog of *gas-1*, NDUFS2 is one of the 14 highly conserved complex I subunits, thought to form the minimal functional core of CO I [10]. Actually, current model suggests that NDUFS2 together with NDUFS3 forms the initial CO I core around which, through a series of assembly steps, functional complex I is formed [10]. Decreased stability and low levels of assembled CO I were detected in a majority of patients with mutation in the NDUFS2 gene [11]. In accordance to data in humans, RNAi mediated downregulation of *gas-1* in worms leads to a harsh decrease in the CO I levels [12]. However, analysis of transcriptional alterations that occur in *gas-1(fc21)* and several other mitochondrial mutants, revealed that many biochemical processes altered in the long-lived Mit mutants were also changed in *gas-1(fc21*) short-lived mutant [13]. Interestingly, some reports on *gas-1(fc21)* mutant indicated that the maximum life span could be extended beyond that of wild type animals, simply by growing them at 15°C [14]. In fact, it was proposed that *gas-1(fc21)* is potentially long-lived mutant that is unable to fully stabilize their MRC supercomplexes and that low temperatures could enhance their supercomplex formation to reveal their latent longevity phenotype [15]. Therefore, as a comparison, we included a long-lived *nuo-6(qm200)* mutant in our analyses. *nuo-6* encodes *C. elegans* homologue of NDUFB4, a peripheral CO I subunits that belongs to a group of so called “single transmembrane domain” (STMD) proteins [16], [17].

We were interested in understanding why the *gas-1(fc21)* mutation leads to life shortening and in analyzing molecular mechanisms shown to underlie the longevity in Mit mutants, in *gas-1(fc21)* animals. Our results indicate that majority of longevity assurance mechanisms are upregulated in *gas-1(fc21)* mutant, similar to *nuo-6(qm200)* long-lived worms. We also show that the stability of CO I plays a major role in determination of longevity and that this can be uncoupled from the formation of reactive oxygen species (ROS) within mitochondria.

## Results

**Figure 1:**
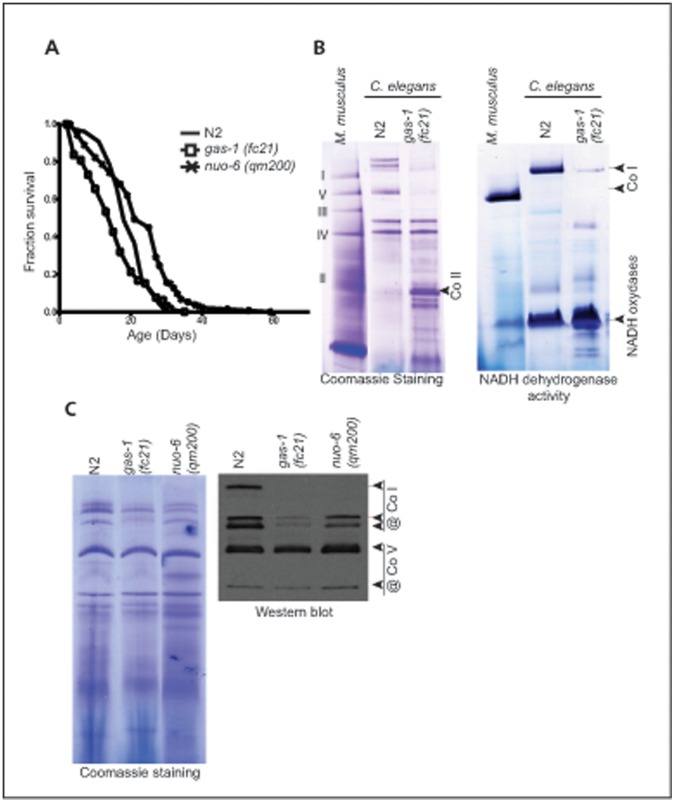
Low levels of mitochondrial complex I in *gas-1(fc21)* mutant. **A** Lifespan analysis of *gas-1(fc21)* (white square) and *nuo-6(qm200)* (black cross) mutants in comparison to wild type (N2, black line) animals. **B** Analysis of mitochondrial complexes in *gas-1(fc21)* mutant. Mitochondrial proteins isolated from mouse liver (M. musculus); wild type worms (N2) or *gas-1(fc21)* mutant were separated on n-dodecyl-β-D-maltoside-based BN-PAGE. The gel was stained with Coomassie Brilliant Blue R-250 solution. **C** Analysis of mitochondrial supercomplexes in Mit mutants. Mitochondrial proteins isolated from wild type worms (N2); *gas-1(fc21)* and *nuo-6(qm200)* mutants were separated on digitonin-based BN-PAGE Gels were stained and *in gel* complex I activity was determined as in B. Detection of the respiratory complexes and supercomplexes obtained after BN-PAGE by western blot using specific antibodies against complex I (CO I) and V (CO V).

Single point mutations in the individual subunits of mitochondrial respiratory complexes (MRCs) can have a strong effect on assembly or stability of the whole complex. NDUFS2 subunit affected in the short-lived *gas-1(fc21)* mutant (Figure 1A) is one of the central subunits and hosts the ubiquinone binding site [16]. The NDUFS2 subunit sits in the peripheral arm of CO I and is also in the contact with the membrane arm [16]. Thus, the functional defects occurring upon mutations in this specific subunit are not surprising at all. The mutation (R290K) affects the central region of GAS-1, which is expected to make contact with one of the membrane integral mtDNA-coded subunits [10]. Thus, mechanistic consequences of *gas-1(fc21)* mutation on the stability of the subunit or the whole CO I are also expected.

We analyzed levels of Complex I on n-dodecyl-β-D-maltoside-based Blue Native Polyacrylamide Electrophoresis (BN-PAGE) (Figure 1B). The level of fully assembled CO I was significantly decreased in the *gas-1(fc21)* mutant (Figure 1B). A number of low molecular-mass bands corresponding to CO I are visualized after staining for the *in gel* NADH activity (Figure 1B), confirming a strong defect in the assembly and/or stability of CO I in the *gas-1(fc21)* mutant. This analysis revealed also presence of much higher levels of other NADH oxidases in worms comparing to mammalian mitochondria (Figure 1B). We also detected high level of complex that correspond in size to mammalian CO II and was not stained for CO I activity (Figure 1B).

Next we analyzed the level and composition of respiratory chain supercomplexes in *gas-1(fc21)* mutants by digitonin-based BN-PAGE (Figure 1C). Using digitonin instead of maltoside will preserve some labile hydrophobic interactions that tie together individual MRC complexes into supercomplexes. We included a long-lived *nuo-6(qm200)* mutant in our analysis of MRC supercomplexes (Figure 1A). G116E mutation found in the *nuo-6(qm200)* mutant is located right at the beginning of the predicted transmembrane helix of the STMD protein and thus likely to interfere with assembly/stability of the Complex I. Interestingly, RNAi mediated downregulation of *nuo-6* gene did not affect the stability of CO I in worms [12]. We observed a strong reduction of MRC supercomplexes in *gas-1(fc21)* mutants, with particular decrease in the amount of the higher molecular weight supercomplexes (Figure 1C). The level of supercomplexes was much more reduced in *gas-1(fc21)* mutants than *nuo-6(qm200)* (Figure 1C). Native Western blots of digitonin-based BN-PAGEs probed with an antibody against CO I NDUFS3 subunit, confirmed these findings (Figure 1C). The amount of Complex V was not changed in different mutants comparing to wild type mitochondria (Figure 1C).

**Figure 2:**
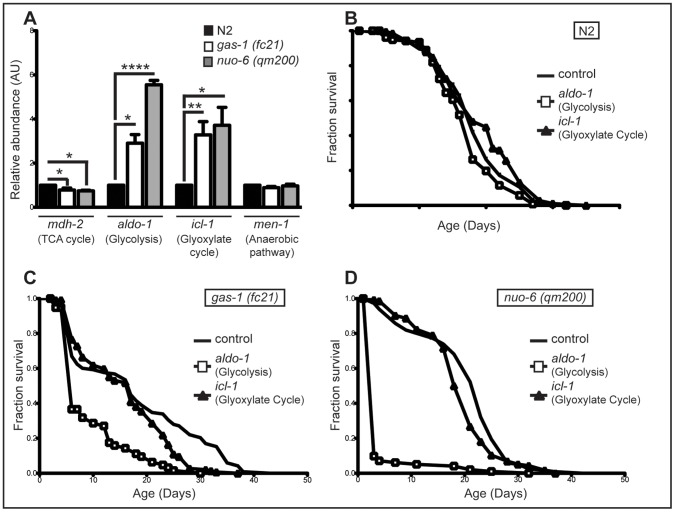
Short and long-lived complex I mutants have similar changes in metabolism. **A** Expression profiles of genes involved in various metabolic pathways: TCA cycle with malate dehydrogenase (*mdh-2*), anaerobic pathways with malic enzyme (*men-1*), glycolysis with fructose 1,6 bisphosphate aldolase (*aldo-1*), glyoxylate cycle with isocitrate lyase/malate synthase (*icl-1*). N2 (black bar), gas-1(fc21) (white bar) and nuo-6(qm200) (grey bar) animals were collected at day 1 of adulthood from synchronized populations. Bars represent relative abundance compared to control; means ± SEM. Asterisks indicate statistical significance in comparison to N2 (Student’s t-test, (*p<0.05, **p<0.01, ***p<0.0001, ****p<0.00001; n = 4). **B–D**. Effect of RNAi knockdown of *aldo-1* and *icl-1* on lifespan in (**B**) with wild type (N2); (**C**) *gas-1(fc21) and* (**D**) *nuo-6(qm200)* worms.

MRC defects in humans often lead to upregulation of alternative metabolic pathways. It has been shown that long-lived Mit mutants also have a change in metabolism, switching from respiratory metabolism, toward alternative, more anaerobic pathways [15]. Therefore, we investigated the expression patterns of key enzymes involved in some of the major alternative metabolic pathways in *C. elegans*. Initially, we used qRT-PCR to measure the expression of genes involved in: tricarboxylate cycle (TCA) - *mdh-2* (mitochondrial malate dehydrogenase), glycolysis - *aldo-1* (Fructose-bisphosphate aldolase), glyoxylate pathway - *icl-1* (isocitrate lyase/malate synthase) and anaerobic pathway – *men-1* (malic enzyme) (Figure 2A). We noticed a slight decrease in the transcript level of *mdh-2*, one of the TCA cycle enzyme in both mutants. However, transcript levels of other measured TCA cycle genes were either slightly upregulated (*fum-1*, fumarase) or unchanged (*ogdh-1*, 2-oxoglutarate dehydrogenase), indicating no general change in the TCA cycle (data not shown). *aldo-1* expression showed a three-fold increase in *gas-1(fc21)* and almost a six-fold increase in *nuo-6(qm200)* mutants (Figure 2A). We detected similar four-fold increased in *icl-1* transcript levels in both mutants (Figure 2A). Although, downregulation of these genes in wild type worms (N2) did not affect the lifespan (Figure 2B), *gas-1(fc21)* mutants showed dependence on both *aldo-1* and *icl-1* (Figure 2C). We detected a strong decrease in *gas-1(fc21*) median lifespan of up to 65% when *aldo-1* was downregulated and a milder effect (15%) when *icl-1* was downregulated (Figure 2C). Correspondingly, presence of *aldo-1* seems to be an absolute prerequisite for the longevity of *nuo-6(qm200)* mutants, as its downregulation drastically decreases median lifespan for more than 85% (Figure 2D). *icl-1* deficiency in the same strain had again much milder effect (Figure 2D). Activation of other anaerobic pathway(s) was not observed measured by the transcript levels of *men-1* in either mutants (Figure 2A). As anticipated from expression data, downregulation of *mdh-1* or *men-1* in either strain did not affect lifespan (data not shown). This data indicate that both *gas-1(fc21*) and *nuo-6(qm200*) rely heavily on glycolysis for energy production and to a lesser extent on the glyoxylate pathway. However, the observed metabolic switch does not provide explanation for the lifespan difference of these mutants.

**Figure 3:**
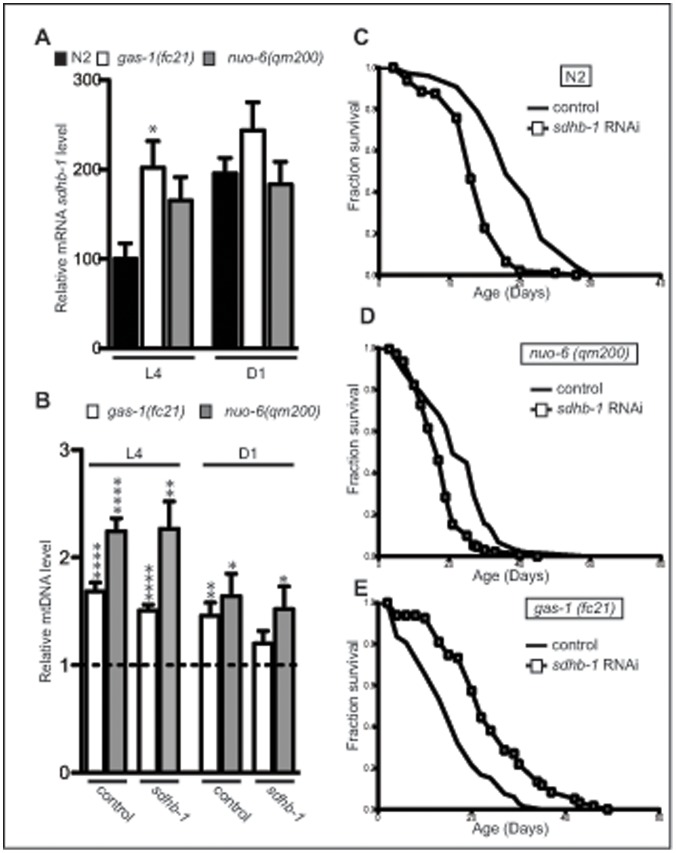
Complex II negatively regulates lifespan in *gas-1(fc21)* mutant. **A** Relative *sdhb-1* transcript levels in wild type (N2), *gas-1(fc21) and nuo-6(qm200)* animals at L4 larval stage (L4) and day 1 (D1) of the adulthood. The bars represent means ± SEM. Asterisks indicate statistical significance in comparison to N2 worms in the control conditions (Student’s t-test, *p<0.05, n = 5). **B** Relative mitochondrial DNA levels in *gas-1(fc21)* and *nuo-6(qm200)* animals compared to wild type (dashed line) with or without *sdhb-1* RNAi. The bars represent mean ± SEM. (n = 6–8) Asterisks indicate statistical significance in comparison to wild type worms in the control conditions (Student’s t-test, *p<0.05; n = 6–8). **C–E** Effect of RNAi knockdown of *sdhb-1* gene on the lifespan of (**C**) wild type (N2); (**D**) *nuo-6(qm200)* and (**E**) *gas-1(fc21)* mutants.

Complex I is the first entry point for electrons into the mitochondrial electron transport chain. CO I catalyzes electron transfer from NADH to quinone coupled to protons translocation across the mitochondrial membrane [18]. CO II is also forming an entry point into the electron transport chain by using succinate, produced in the TCA cycle, as a substrate. It is not surprising that often, downregulation of CO I subunits leads to an upregulation of SDH activity, probably to increase entry of electrons into respiratory chain [18]. The upregulation of CO II dependent respiration was also one of the compensatory mechanisms observed in the *gas-1(fc21*) mutants [14]. We confirmed these results with a technique that we recently adapted to visualize and quantify MRC activities *in situ,* on the level of single tissues by using histochemical staining of fresh frozen *C. elegans* sections (Figure S1A and [19]). We detected 4–6 times upregulation in the SDH activity in *gas-1(fc21*) mutant in all investigated tissues (intestine, body wall muscle and pharynx) [19]. We also observed strong upregulation of CO II levels in *gas-1(fc21*) mutants on BN-PAGE used to visualize single MRC complexes (Figure 1B). A two-fold upregulation of *sdhb-1* transcript levels was detected at L4 larval stage, but was normalized later in life (Figure 3A). Therefore, high CO II levels during adulthood are likely maintained by increased stability/decreased turnover of CO II subunits. In addition, an upregulation of Cytochrome c Oxidase (COX) activity was also detected in these worms, but to a much lesser extent [19]. Increase in COX and SDH activity is indicator of mitochondrial stress and suggest upregulation of mitochondrial mass. We measured the mtDNA levels that give a pretty good indication of the mitochondrial mass within a cell. We detected around 50% more mtDNA in both *gas-1(fc21)* and *nuo-6(qm200)* strains at D1 of adulthood and even higher increase at L4 stage specifically in *nuo-6(qm200)* mutants, than measured in N2 control worms in the same conditions (Figure 3B). Taken together these results show that there is a certain increase in mitochondrial biogenesis (≈50%) that cannot fully account for the observed increase in SDH activity in *gas-1(fc21)* mutant (400–600%) [19], indicating significant upregulation of CO II levels per individual mitochondrion. In the light of this, we tested if and to what extent CO II levels and activity are important for the lifespan determination in *gas-1(fc21)* and *nuo-6(qm200)* mutants.

CO II usually consists of four subunits (SDHA-D) needed to channel electrons coming from the oxidation of succinate to fumarate, further to ubiquinone. Two peripheral subunits (SDHA-B) also work as the enzyme succinate dehydrogenase (SDH) of the tricarboxylic acid cycle (TCA). We investigated whether inactivation of complex II by RNAi-mediated suppression of two peripheral SDH subunits (*sdha-1, sdha-2, sdhb-1* genes in *C. elegans*) would impact the lifespan of *gas-1(fc21)* and *nuo-6(qm200)* mutants. *C. elegans*, like the parasitic nematode *Ascaris suum* contains two complex II flavoprotein (Fp) subunits (*sdha-1 and sdha-2*) [20]. No difference in the life span of either strain (N2, *gas-1(fc21)* or *nuo-6(qm200)*) was observed after the inactivation of *sdha-1* and *sdha-2* subunit (Figure S2). This does not come as a surprise, as the two genes were shown to be functionally redundant and important under aerobiosis [8]. Unexpectedly, down-regulation of the *sdhb-1* subunit, had completely opposite effect on the lifespan of *gas-1(fc21)* and *nuo-6*(*qm200)* mutants (Figure 3C and 3E). While downregulation of *sdhb-1* led to a decrease in mean and maximal lifespan of N2 and *nuo-6*(*qm200)* mutant (Figure 3C and 3D, respectively), general increase in the lifespan, even beyond normal wild type level, was detected in *gas-1(fc21)* animals upon *sdhb-1* knockdown (Figure 3E and Table S1). A prolongation of the *gas-1(fc21)* lifespan was also observed upon *sdhc-1* and *sdhd-1* knockdown (Table S1).

**Figure 4:**
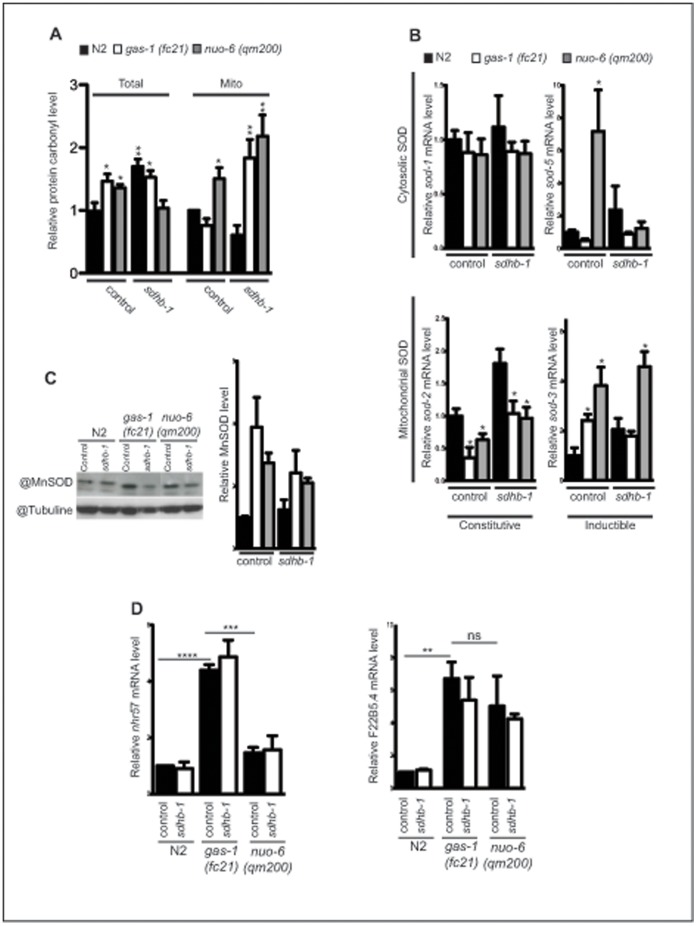
*Sdhb-1* mediates *gas-1(fc21)* longevity independently of ROS production and/or activation of Hif-1 pathway. **A** Relative abundance of carbonylated proteins in total or mitochondrial extract isolated from wild type (N2), *gas-1(fc21)* and *nuo-6(qm200)* animals (n = 4–5). **B–C** SOD transcript and protein levels measured by (**B**) quantitative real-time RT-PCR and (**C)** Western blot. The relative abundance of SOD2 protein is shown on the lower C panel. (n = 4). **D** Transcript levels of HIF-1α-responsive genes *nhr-57* and *F22B5.4* in wild type (N2), *gas-1(fc21)* and *nuo-6(qm200)* animals. Data were obtained from worms collected from synchronized population at day 1 of adulthood. The worms were grown either on control plates (L4440) or on *sdhb-1* RNAi plates (n = 5). In all panels bars represent mean ± SEM. Asterisks indicate statistical significance in comparison to wild type worms in the control conditions (Student’s t-test, *p<0.05, **p<0.01; ***p<0.001).

*Gas-1(fc21)* animals are extremely sensitive to increased oxidative tension or exposure to paraquat [14]. Increased ROS production as well as high levels of protein carbonyls and 4-hydroxy-2-nonenal (HNE) adducts have been detected in mitochondria isolated from *gas-1(fc21)* animals [21]. CO II was implicated in both ROS production and defence against oxidative stress through its specific role in the maintenance of the mitochondrial UQ pool [22]. Therefore, we tested if downregulation of *sdhb-1* influences oxidative stress levels by measuring protein carbonyls in the whole cell lysates of *gas-1(fc21)* or *nuo-6(qm200)* mutants (Figure 4A). Quantification of the resulting Western blots revealed that the oxidative damage in both mutants is higher than in N2 worms (Figure 4A). Upon silencing of the *sdhb-1* subunit, we observed an increase in protein carbonyl levels in wild type worms, while in *gas-1(fc21)* mutants level of protein carbonyls stayed equally upregulated (Figure 4A). We observed a significant decrease in oxidative damage in *nuo-6(qm200)* strain under the same conditions (Figure 4A). When the same analysis was performed on isolated mitochondria we obtained different results (Figure 4A). *sdhb-1* downregulation in *gas-1(fc21)* mutant led to strong upregulation of oxidative stress. As this upregulation does not affect overall levels of oxidative stress it is likely that the ROS responsible for this damage is produced inside mitochondrial matrix, most probably at the level of CO I. Similar results were obtained with *nuo-6(qm200)* mutant (Figure 4A). In this case, higher level of CO I containing mutated subunit might increase CO I ROS production already on control plates (Figure 4A).

We next analyzed expression levels of superoxide dismutases (SOD), which are the first line of antioxidant defence against ROS generated during respiration by converting superoxide to hydrogen peroxide. *C. elegans* has five *SOD* genes that can be divided in two basic categories: primary *(sod-1*, *sod-2* and *sod-4)* and inducible *(sod-3* and *sod-5)* [23]. Normal levels of *sod-1* transcripts were found in all different strains and in all different conditions (Figure 4B). We detected lower *sod-2* transcript levels in both *gas-1(fc21)* and *nuo-6(qm200)* mutant in normal conditions that were normalized upon *sdhb-1* silencing (Figure 4B). In the same time SOD-2 protein levels were slightly upregulated or unchanged in these samples (Figure 4C). Both inducible SODs were highly upregulated in the *nuo-6(qm200)* strain, with *sod-5* levels returning to normal upon *sdhb-1* silencing (Figure 4B). Results from *gas-1(fc21)* strain basically mirrored previous results: we observed slight upregulation of *sod-3*, but not *sod-5* levels in untreated worms, and these did not changed after *sdhb-1* RNAi treatment (Figure 4B).

Another pathway recently recognized to be involved in promoting longevity upon inhibition of respiration is through activation of hypoxia-inducible factor (HIF-1α) [24]. The HIF-1 is a highly conserved transcription factor, initially identified as activator of specific stress genes that promotes survival during hypoxia [25]. Although increase in mitochondrial ROS production has been clearly involved in the HIF-1α stabilization [25], recent work has also identified succinate and fumarate as potent inhibitors of prolyl-4-hydroxylases, thus leading to the stabilization of HIF-1α [26]. Therefore, we tested if the observed longevity of *gas-1(fc21)* mutant, upon silencing of *sdhb-1,* is a result of activation of the HIF-1 pathway through changes in succinate and/or fumarate levels. We measured transcript levels of two *C. elegans hif-1*-dependent hypoxia-inducible genes, *nhr-57* and *F22B5.4*, both of which are shown to be significantly upregulated in other long-lived mitochondrial mutants (*clk-1* and *isp-1* mutants, [24]). The expression of both of these genes was increased in *gas-1(fc21)*, but only levels of *F22B5.4* were upregulated in the *nuo-6(qm200)* mutant (Figure 4D). Contrary to expected, silencing of *sdhb-1* did not have an effect on expression of either *nhr-57* or *F22B5.4* genes (Figure 4D). These results therefore indicate that, neither ROS upregulation nor activation of HIF-1 pathway, determine a switch in longevity of *gas-1(fc21)* mutant upon decrease in CO II levels.

Recent studies showed that in response to a mitochondrial perturbation a specific stress response mechanism is activated to increase the expression of mitochondrial associated protein chaperones, such as HSP-6 or HSP-60. This mechanism is referred to as the mitochondria-specific unfolded protein response (UPR^mt^) [27], [28], [29]. Disruption of MRC subunits either by RNAi silencing or by introduced mutation(s) activates this mitochondrial stress response, proposed to be essential for the longevity induced by mitochondrial dysfunction [30]. We tested whether UPR^mt^ is differentially activated in short and long – lived mutants (*gas-1(fc21)* and *nuo-6(qm200)*, respectively) and if this stress response is affected by downregulation of CO II through *sdhb-1* silencing. We monitored the UPR^mt^ by following the fluorescence of *hsp-6p::GFP*, a specific marker of the UPRmt pathway (Figure S3). An upregulation of UPR^mt^ was detected in *nuo-6(qm200)* (Figure S3), as previously demonstrated in other long-lived Mit mutants [30]. We also observed an activation of the UPR^mt^ signal in *gas-1(fc21)* mutant (Figure S3), and this has not changed with a switch in longevity, observed upon downregulation of CO II (data not shown). Judging by the intensity of the GFP signal, it seems to be that *gas-1(fc21)* mutant upregulates UPR^mt^ to a higher degree than *nuo-6(qm200)*, indicating that the mitochondrial protein homeostasis is affected more by *gas-1(fc21)* mutation. This is in accordance with our data on stability of CO I and supercomplexes in these two mutants.

**Figure 5:**
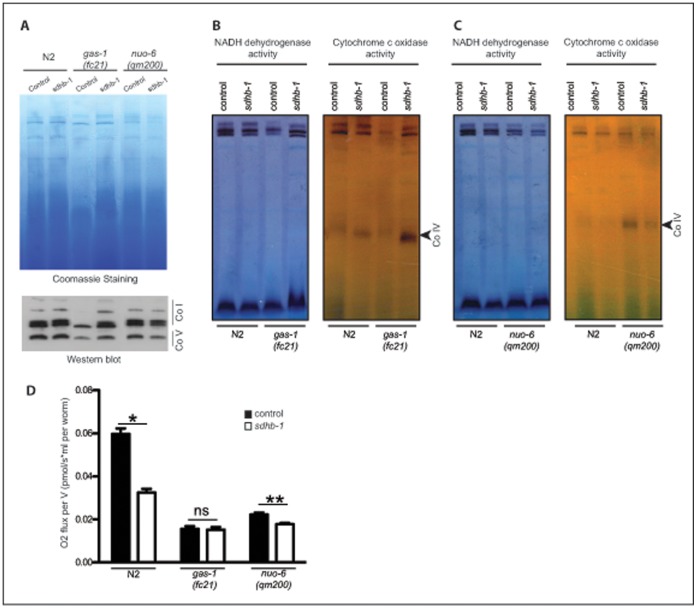
*Sdhb-1* silencing stabilizes CO I levels in *gas-1(fc21) mutant.* **A** Analysis of mitochondrial supercomplexes in wild type worms (N2); *gas-1(fc21)* and *nuo-6(qm200)* mutants grown either on control plates (L4440) or on *sdhb-1* RNAi plates (n = 5). Mitochondrial proteins were separated on digitonin-based BN-PAGE. Detection of the respiratory complexes and supercomplexes obtained after BN-PAGE by western blot using specific antibodies against complex I (CO I) and V (CO V). **B** and **C** *In gel* analysis of NADH dehydrigenase and Cytochrome c Oxidase activity was performed after BN-PAGE to reveal the level of active complex I and IV, respectively by using (**B**) *gas-1(fc21)* and (**C**) *nuo-6(qm200)* animals. **D** Oxygen consumption of wild type worms (N2); *gas-1(fc21)* and *nuo-6(qm200)* animals subjected to RNAi against *sdhb-1* at day 1 of adulthood. The bars represent means ± SEM. Asterisks indicate statistical significance in comparison to N2 worms in the control conditions (Student’s t-test, *p<0.05, **p<0.01, n = 5).

Mutual interdependency of CO I, III and IV in respiratory supercomplexes is clearly established [31]. It is far less clear what is complex II role in this and what would be the consequences of its upregulation on the stoichiometry of supercomplexes. There is an ongoing debate if CO II is associated with supercomplexes or not [32], [33]. We speculated that the increase in CO II levels could have a disadvantageous effects on the stability of CO I in *gas-1(fc21)* mutants. Therefore, we analyzed levels of MRC supercomplexes by the digitonin-based BN-PAGE, before and after *sdhb-1* knockdown (Figure 5A–C). Indeed, we noticed that a reduction in the *sdhb-1* level leads to an increase in the amount of CO I, only in the *gas-1(fc21)* mutant background (Figure 5A and 5B). The same intervention had little, if any effect in the wild type or *nuo-6(qm200)* mutant (Figure 5A and 5C). This has been confirmed by the analysis of CO I and CO IV *in gel* activity (Figure 5B and 5C) and by Western blotting with antibody against CO I and CO V subunits (Figure 5A).

We propose that CO II level, not the SDH activity is a primary suppressor of the longevity in *gas-1(fc21)* mutant. We show that 25 mM malonate decreases lifespan of *gas-1(fc21)* mutant to the same level as in wild type or long-lived mitochondrial mutants (Table S2). Lower concentrations of malonate (2 mM) did not have an obvious effect on the lifespan (Figure S1C). In order to understand the level of CO II suppression by malonate treatment vs. *sdhb-1* knockdown we estimated CO II activity by staining fresh-frozen sections of N2 and *gas-1(fc21)* mutants grown either on control plates, plates with 25 mM malonate or on RNAi plates against *sdhb-1*. A clear decrease in the SDH activity in *gas-1(fc21)* mutants could be detected on plates with 25 mM malonate and even higher suppression on *sdhb-1* RNAi plates. We believe that a decrease detected in worms grown on plates with malonate could be underestimated as we observed that washing steps often remove non-covalently bound malonate from sections. In agreement, we detected much higher decrease in SDH activity when we added 25 mM malonate directly to the staining solution (Figure S1B). Furthermore, we monitored lifespan in relation to malonate concentration present in the plates. We detected an apparent decrease of median lifespan with increasing concentrations of malonate in all strains (Figure S1C). Our data demonstrate that comparable decrease in CO II activity has a very different effect on median lifespan (malonate ≈5 days, sdhb-1 RNAi ≈22 days) of *gas-1(fc21)* mutants depending if CO II levels are changed or not. In agreement with this, it was shown that double mutants carrying mutations in both CO I (*gas-1(fc21))* and CO II (*mev-1(kn1))* genes are synthetic lethal 14.

Beside increase in lifespan, a positive effect of lowering CO II levels was also observed through normalization of mtDNA levels in *gas-1(fc21)* mutants, indicating decrease in mitochondrial mass (Figure 3B). Furthermore, in contrast to N2 and *nuo-6(qm200)*, *gas-1(fc21)* respiration did not change upon *sdhb-1* downregulation despite high reduction of CO II activity (Figure 5D). This suggests that increase in respiration comes from the higher electron flow through CO I.

## Materials and Methods

### Nematode Strains

Wild type (*C. elegans*, N2, Bristol), as well as the mutants *gas-1(fc21)* and *nuo-6(qm200)* were obtained from the *Caenorhabditis* Genetics Center in Minneapolis. Standard techniques were used for growing and maintaining cultures of *C. elegans* [34].

### Lifespan Assay

To inhibit specific gene function and examine its influence on the lifespan we used a standard feeding RNAi protocol [35]. The effect of inhibition of the following genes was examined: F42A8.2 (referred to *sdhb-1*), *aldo-1* (T05D4), *icl-1* (C05E4), *men-1* (Y48B6A). The RNAi effect was followed during entire lifespan. All RNAi clones were retrieved from the Ahringer RNAi library and checked before their use by sequencing. Worms fed on bacteria carrying the empty vector (L4440) were used as a control. Synchronized populations were used for lifespan measurements. These populations were obtained by bleaching of worm strains and starting the culture from the resulting isolated embryos [36]. At least 100 animals were used per condition and scored every or every other day. Worms that died due to internally hatched eggs, vulva protrusion, and desiccation or due to crawling out of the plate were censored. All lifespan assays were conducted at 20°C. The cumulative survival rate was determined according to Kaplan and Meier [37]. The log-rank (Mantel–Cox) test was used for comparing significant population distributions differences between different groups in the lifespan assays. Data analysis was performed in GraphPad Prism 5 software.

### Measurement of Transcript Levels

Transcript levels of genes involved in different metabolic pathways were analyzed in hermaphrodite animals by quantitative real-time PCR. Total RNA was isolated with Trizol (Invitrogen) and quantified by spectrophotometry. 0.8 µg of total mRNA was reversely transcribed using High Capacity cDNA Reverse Transcription Kit (Applied Biosystems, USA). Each sample was obtained with 3 to 5 independent preparations and analyzed at least two times. Real time PCR was performed by the ABI Prism 7900Fast Sequence Detector (Applied Biosystems) with the following PCR conditions: 3 min at 95°C, followed by 40 cycles of 5 sec at 95°C and 15 sec at 60°C. Amplified products were detected with SYBR Green (Brilliant III Ultra Fast SYBR Greeb qPCR Master Mix, Agillent Technologies). Relative quantification was performed against actin. Standard curves were constructed by using four subsequent 10-fold dilutions of the reference sample. Standards were run in triplicates with actin (*act-3*) as endogenous control. Primers used for these analysis are presented in Table S3. Data analysis was performed with GraphPad Prism5 software.

### Determination of mtDNA Copy Number

The mtDNA copy number was measured by quantitative PCR as previously described [38]. Worms at the day 1 of adulthood were singled and lysed by standard protocol [38]. Quantitative PCR was performed for at least 4 independent samples. Data analysis was performed with GraphPad Prism5 software.

### Measurement of Carbonylated Proteins

Protein carbonyl groups were detected and quantified with the OxyBlot protein oxidation detection kit (Chemicon International). In brief, 50 µg or 30 µg of total cell extract or isolated mitochondria, respectively were used for the experiments where carbonylated proteins were measured using 2,4-dinitrophenyl hydrazine (DNPH) antibodies. Each fraction was treated with DNPH as described by [39] with some modifications [40]. Protein concentrations were determined using Bradford assay. For Western blot analysis, samples were transferred to Polyvinylidene fluoride (PVDF) membranes using an iBlot system (Invitrogen, Carlsbad, CA). Immunoreactive proteins were visualized using the enhanced chemiluminescence system (ECL, Amersham Biosciences, Uppsala, Sweden).

### Isolation of Mitochondria

Mitochondria were isolated from wild type (N2), gas-1(fc21) and nuo-6(qm200) strains. The strains were synchronized by hypochlorite treatment and cultivated on RNAi plates seeded with bacteria carrying an empty vector (L4440) or the *sdhb-1* RNAi vector. Worms were collected at day 1 of adulthood with S-basal [34]. One gram of worm pellet (wet mass) was resuspended in 10 ml of MSE buffer (220 mM mannitol, 70 mM sucrose, 10 mM Tris, 2 mM EDTA, pH 7.4). Worms were homogenized with an engine-driven potter (500 rpm×5 times). After homogenization, subtilisin A was added (10 mg/g of worm pellet) and worm suspension was incubated for 20 min at 28°C, followed by additional manual homogenization. Subcellular fractionation to isolate mitochondria was performed as follows: the homogenate was spun down at 10 000×g for 5 min at 4°C, resuspended in 10 ml of MSE buffer, 0.4% BSA (fatty acids free), and spun again at 1 000×g for 5 min at 4°C to remove cellular debris. Mitochondria were retrieved from the supernatant by centrifugation at 10 000×g for 5 min at 4°C and resuspended in MSE buffer. Protein concentration was determined using Bradford’s method (absorption at 595 nm).

### Blue Native Electrophoresis

Blue Native gel electrophoresis (BN-PAGE) was performed as previously described [41]. In brief, electrophoresis was performed using a minigel system (Xcell SureLock Mini Cell, Invitrogen) and commercial ready-to-use blue native gels (Native–PAGE Novex Bis–Tris Gel system, 3–12% gel; Invitrogen). Mitochondrial proteins (50 µg) were solubilised by digitonin using the detergent/protein mass ratio of 6/1, or solubilised in 1.0% of n-dodecyl-β-D-maltoside. The gels were stained with Coomassie Brilliant Blue R-250 solution according to the standard procedure [42]. For detection of dehydrogenase activity characteristic for CO I, *in gel* activity assays were performed by incubating gels for at least 1 h at room temperature with 5 mM Tris–HCl, pH 7.4, 0.1 mg/ml NADH, and 2.5 mg/ml NTB (nitrotetrazolium blue, Sigma). Western blotting of BN-PAGE gels was essentially performed according to the manufacturers procedure using PVDF blotting membranes and iBlot system (Invitrogen, Carlsbad, CA). For detection of CO I, a monoclonal antibody against human NDUFS3 (homologue of *C. elegans* NUO-2) was used (MS112, mouse, MitoSciences) at a dilution of 1∶1000. For detection of CO V, a monoclonal antibody against ATP synthase subunit alpha was used (MS507, mouse, MitoSciences) at a dilution of 1∶1000.

## Discussion

Our results show that mitochondrial dysfunction, even in a short-lived mutant like *gas-1(fc21),* activates longevity assurance program that include alternative metabolic and stress pathways. However, these pathways on their own cannot ensure lifespan prolongation that seems to be dependent on the level and type of mitochondrial dysfunction. We show that in *gas-1(fc21)* mutant mitochondrial function is below a threshold needed for the lifespan prolongation. Increased stability of CO I seems to improve mitochondrial function and pushes the balance toward increase in longevity.

With a very few exceptions, knockdown of most CO I subunits, including *gas-1* and *nuo-6* genes leads to upregulation of CO II-dependent OXPHOS activity in worms, [12]. Thus it is not completely clear why this has a deleterious effect only on *gas-1(fc21)* mutants. A possible explanation may lay in the fact that GAS-1 (NDUFS2) and NUO-6 (NDUFB4) have a very different localization and function in CO I. While NDUFS2 carries a ubiquinone binding site and forms the catalytic core of CO I, NDUFB4 is a peripheral subunit of CO I that is associated with both subcomplex Iα and Iβ [16], [43]. Mutation or loss of both of these genes leads to decreased stability of CO I. Compensatory upregulation of CO II that is probably present in both strains, leads to further depletion of CO I only in *gas-1(fc21)* mutants likely by decreasing ubiquinone pools. It is well known that ligands can promote protein folding and stabilization and improved quinone binding to GAS-1 subunit could therefore increased CO I stability. In fact, it has been shown that mutations in C. elegans nuo-1 gene (NDUFV1 gene in mammals), encoding the 51-kDa CO I subunit that carries the FMN cofactor cause marked depletion of both CO I and CO IV [44]. Supplementation with riboflavin, a precursor to the flavin cofactors, likely results in increased FMN availability, leading to enhanced rates of NUO-1 folding to a more stable cofactor-bound form and to assembly into stable MRC complexes [44]. We also believe that only in combination with a specific CO I defect, increased CO II levels could have a deleterious effect as seen in *gas-1(fc21)* mutants. Alternative explanation for the observed phenotypes could be that upon increased expression, significant portion of CO II gets incorporated into supercomplexes decreasing chances of already unstable CO I to be “embraced” and therefore stabilized by interaction with CO III and IV. This is however less likely as it seems that majority of the CO II is not part of MRC supercomplexes [45].

The inhibition of distal CO II subunits (SDHB – D), either pharmacologically or via RNA interference, increases normoxic ROS production and hypoxia-inducible factor 1 alpha (HIF-1α) stabilization in a ROS-dependent manner, and this leads to an increase in growth rates of mammalian cells *in vitro* and *in vivo* [22]. Accordingly, point mutation in the quinone-binding pocket of SDHC (*mev-1*) subunit of CO II also increases oxidative stress and superoxide anion production [46]. Furthermore, increased ROS production and upregulation of HIF-1α stress pathway were shown to be essential for lifespan prolongation in long-lived Mit mutants, including *nuo-6* [24], [47]. Indeed, our results clearly demonstrate that mitochondrial dysfunction in *gas-1(fc21)* animals increases the oxidative damage to the cell and activates HIF-1α stress pathway. This does not, however, change upon downregulation of *sdhb-1* subunit demonstrating that the HIF-1α longevity assurance pathway is already in place, but is not sufficient to ensure lifespan prolongation. Similarly, we showed that another longevity assurance stress response, the UPR^mt^, is equally activated in both short-lived and long-lived mutants and therefore can be uncoupled from their longevity. Situation is far less clear in *nuo-1(ua1)* mutants: although we did not observed a change in HIF-1α pathway activation upon *sdhb-1* downregulation, the overall oxidative stress levels showed direct correlation with longevity in agreement with previous studies [24], [47].

A question remains: why increased amounts of assembled CO I in *gas-1(fc21)* animals lead to the lifespan prolongation? We show that increase in CO I stability leads to an increase in stability of supercomplexes and this improves mitochondrial function and decreases mitochondrial stress. We believe that this shifts the balance toward a limited “window” of mitochondrial dysfunction, allowing the lifespan prolongation. Higher CO I levels could increase membrane potential that, in turn will have a positive effect on many cellular processes that depend on iron sulphur cluster synthesis, timely delivery of various metabolic precursors or some other essential mitochondrial function independent of oxidative phosphorylation.

Many studies of the last 50 years showed that mitochondria are involved in the regulation of physiological aging. However, the underlying mechanisms are still unknown. We think that with this study we have come a step further showing that mitochondrial dysfunction activates different longevity assurance pathways that hold a promise of increased longevity. Level and type of mitochondrial dysfunction is what actually decides the animal fate and this seems to be different and unique for each Mit mutant.

## Supporting Information

Figure S1 SDH staining of fresh-frozen sections of wild type (N2) and *gas-1(fc21)* animals. **A** Sectioning was performed on synchronized population subjected to malonate (25 mM) or RNAi against *sdhb-1*. at day 1 of adulthood. **B** SDH staining of fresh frozen wild type animals with direct addition of malonate (25 mM) in staining solution **C** Effect of different concentrations of malonate on median lifespan of wild type (N2); *gas-1(fc21)* and *nuo-6(qm200)* animals.
(PDF)

Figure S2 Effect of RNAi knockdown of the *sdha-1* and *sdha-2* subunit on lifespan. Life span analysis of wild type (N2), *gas-1(fc21)* and *nuo-6(qm200)* worms growing on control plates (L4440) or on RNAi plates for *sdha-1* or *sdha-2* subunits of complex II.
(TIF)

Figure S3 UPR^mt^ response in Mit mutants. UPR^mt^ response was followed via phsp-6::GFP expression in wild type; *nuo-6(qm200)*, *gas-1(fc21)* mutant, or in animals grown on *sdhb-1* RNAi plates. Images are taken at the L3 stage and day 1, day 5 and day 10 of adulthood.
(PDF)

Table S1 Lifespan analyses.
(DOCX)

Table S2 Effect of different electron transport chain inhibitors on the lifespan of *gas-1(fc21)* and *mev-1(kn1)* mutants.
(DOCX)

Table S3 RT-PCR primers.
(DOCX)
