# Supplementary figures and images for: Succinate Dehydrogenase Upregulation Destabilize Complex I and Limits the Lifespan of gas-1 Mutant
Source: PLoS One. 2013 Mar 28;8(3):e59493. doi: 10.1371/journal.pone.0059493 (PMC3610896; doi:10.1371/journal.pone.0059493)

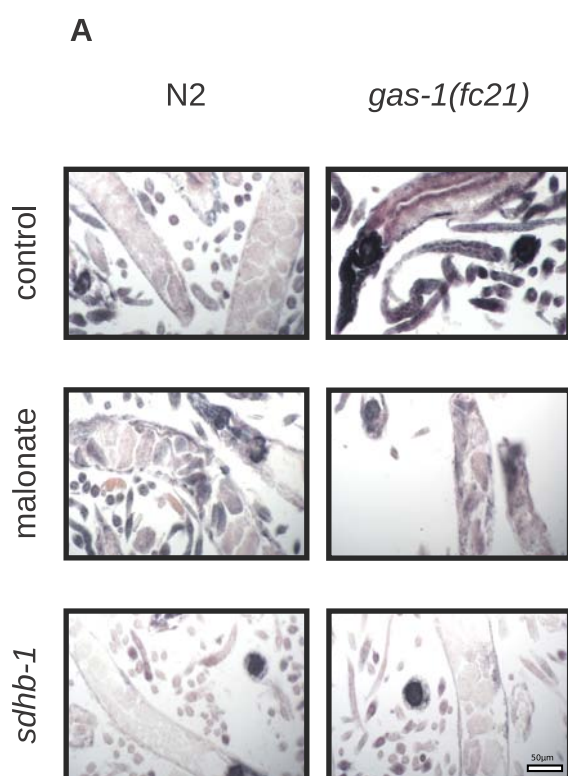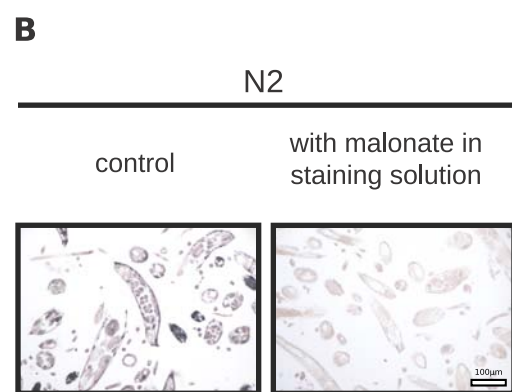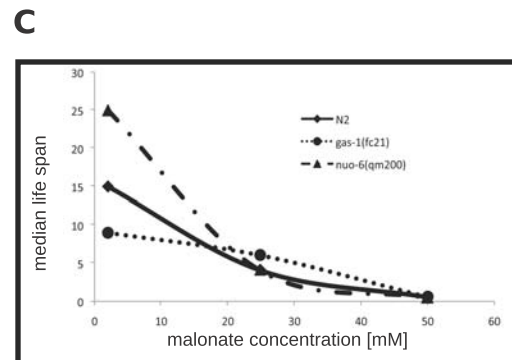

Supplement: Figure S1 — SDH staining of fresh-frozen sections of wild type (N2) and gas-1(fc21) animals. A Sectioning was performed on synchronized population subjected to malonate (25 mM) or RNAi against sdhb-1. at day 1 of adulthood. B SDH staining of fresh frozen wild type animals with direct addition of malonate (25 mM) in staining solution C Effect of different concentrations of malonate on median lifespan of wild type (N2); gas-1(fc21) and nuo-6(qm200) animals. (PDF) [file pone.0059493.s001.pdf]

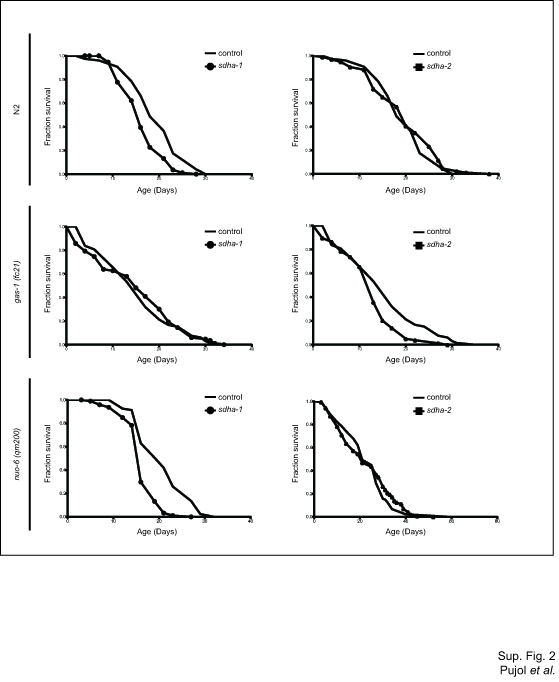

Supplement: Figure S2 — Effect of RNAi knockdown of the sdha-1 and sdha-2 subunit on lifespan. Life span analysis of wild type (N2), gas-1(fc21) and nuo-6(qm200) worms growing on control plates (L4440) or on RNAi plates for sdha-1 or sdha-2 subunits of complex II. (TIF) [file pone.0059493.s002.tif]

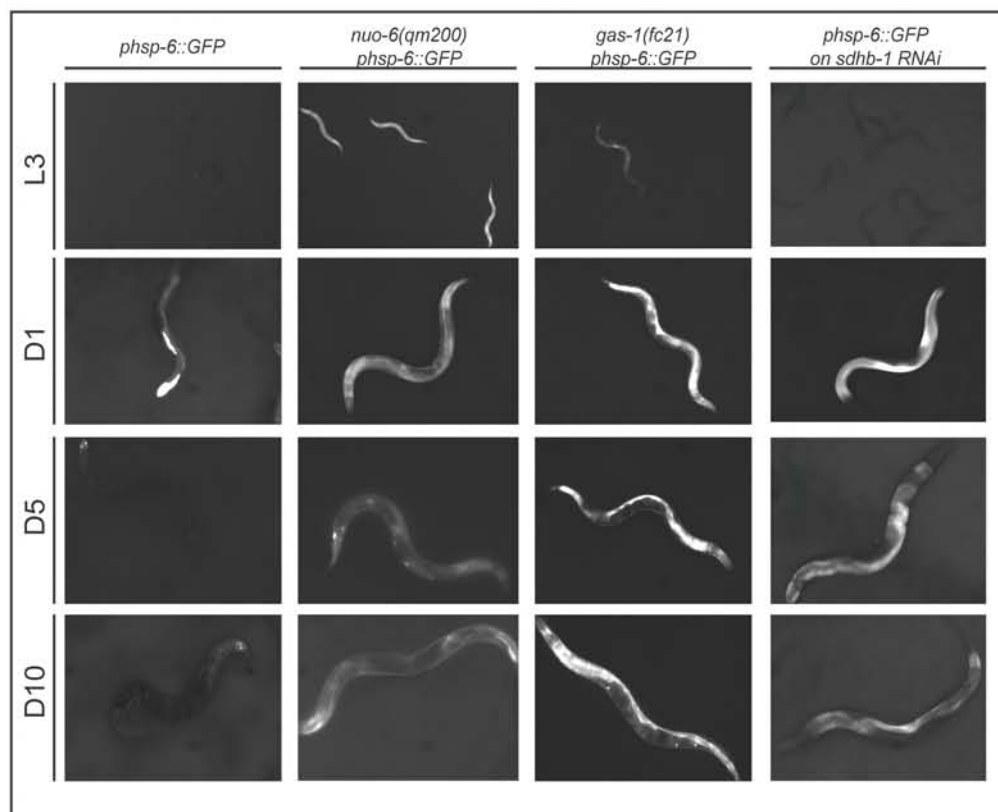

Supplement: Figure S3 — UPRmt response in Mit mutants. UPRmt response was followed via phsp-6::GFP expression in wild type; nuo-6(qm200), gas-1(fc21) mutant, or in animals grown on sdhb-1 RNAi plates. Images are taken at the L3 stage and day 1, day 5 and day 10 of adulthood. (PDF) [file pone.0059493.s003.pdf]
