# Supplementary material for: Succinate Dehydrogenase Upregulation Destabilize Complex I and Limits the Lifespan of gas-1 Mutant
Source: PLoS One. 2013 Mar 28;8(3):e59493. doi: 10.1371/journal.pone.0059493 (PMC3610896; doi:10.1371/journal.pone.0059493)
Supplement: Table S1 — Lifespan analyses. (DOCX) [file pone.0059493.s004.docx]

| Strain | Number of animals that died/total | Median | p-value (log-rank test)* | significance |
| --- | --- | --- | --- | --- |
| N2-L4440^a^ | 74/100 | 18 |  |  |
| N2-*sdha1* | 85/100 | 20 | < 0.0001 | **** |
| N2-*sdha2* | 87/100 | 13 | 0.4595 |  |
| N2-*sdhb1* | 93/100 | 16 | < 0.0001 | **** |
| N2-*sdhc1* | 93/100 | 13 | < 0.0001 | *** |
| N2-*sdhd1* | 79/88 | 18 | < 0.0001 | *** |
| N2-T05D4.1 | 127/180 | 18 | 0.0604 |  |
| N2-C05E4.9 | 184/230 | 18 | 0.0285 | * |
| *gas-1(fc21)*-L4440^b^ | 68/100 | 15 | 0.0166 | * |
| *gas-1(fc21)-sdha1* | 86/100 | 15 | 0.9986 |  |
| *gas-1(fc21)-sdha2* | 85/100 | 13 | 0.0042 | ** |
| *gas-1(fc21)-sdhb1* | 64/100 | 22 | < 0.0001 | **** |
| *gas-1(fc21)-sdhc1* | 69/91 | 22 | < 0.0001 | *** |
| *gas-1(fc21)-sdhd1* | 90/100 | 20 | 0 | *** |
| *gas-1(fc21)*-T05D4.1 | 85/200 | 6 | 0.0072 | ** |
| *gas-1(fc21)*-C05E4.1 | 157/200 | 17 | 0.0064 | ** |
| *nuo6(qm200)*-L4440^c^ | 140/200 | 21 | < 0.0001 | **** |
| *nuo6(qm200)-sdha1* | 134/200 | 21 | 0.005 | ** |
| *nuo6(qm200)-sdha2* | 152/200 | 21 | 0.4277 |  |
| *nuo6(qm200)-sdhb1* | 138/200 | 17 | < 0.0001 | **** |
| *nuo6(qm200)-sdhc1* | 125/200 | 16 | < 0.0001 | **** |
| *nuo6(qm200)-sdhd1* | 145/200 | 17 | < 0.0001 | **** |
| *nuo6(qm200)*-T05D4.1 | 148/160 | 3 | < 0.0001 | **** |
| *nuo6(qm200)*-C05E4.9 | 119/160 | 18 | 0.027 | * |

* Log-rank (Mantel-Cox) Test
