# Supplementary material for: Succinate Dehydrogenase Upregulation Destabilize Complex I and Limits the Lifespan of gas-1 Mutant
Source: PLoS One. 2013 Mar 28;8(3):e59493. doi: 10.1371/journal.pone.0059493 (PMC3610896; doi:10.1371/journal.pone.0059493)
Supplement: Table S2 — Effect of different electron transport chain inhibitors on the lifespan of gas-1(fc21) and mev-1(kn1) mutants. (DOCX) [file pone.0059493.s005.docx]

|  | **N2** | | ***gas-1(fc21)*** | | ***mev-1(kn1)*** | |
| --- | --- | --- | --- | --- | --- | --- |
|  | Mean±SEM | Median | Mean±SEM | Median | Mean±SEM | Median |
| control | **14.55±0.92** | **16** | **10.97±0.42** | **11** | **6.98±0.47** | **8** |
| Rot 25μM* | **4.82±0.34** | **4** | **1±0** | **1** | **4.23±0.61** | **3** |
| Mal 25μM* | **5.75±0.8** | **5** | **4,97±+0.62** | **4** | **3.36±0.41** | **5** |

*rot = Complex I inhibitor rotenone, mal = Complex II inhibitor malonate.
