# Supplementary material for: Succinate Dehydrogenase Upregulation Destabilize Complex I and Limits the Lifespan of gas-1 Mutant
Source: PLoS One. 2013 Mar 28;8(3):e59493. doi: 10.1371/journal.pone.0059493 (PMC3610896; doi:10.1371/journal.pone.0059493)
Supplement: Table S3 — RT-PCR primers. (DOCX) [file pone.0059493.s006.docx]

| Pathway  Gene name | | Forward primer | Reverse primer |
| --- | --- | --- | --- |
|  | | |  |
| **Glycolysis** | | | |
| Fructose 1,6 bisphosphate aldolase | T05D4.1 | GCACAAGATCTCATCTACGACTCC | GAGAGCGTGGTACACATATGAGAG |
| **Citric Acid Cycle** | | | |
| Malate dehydrogenase (organelle) | F20H11.3 | TTCCGAGCTTAAGGGACATGAC | GAGAATTTGGTGGATGGTTTGAC |
| **Glyoxylate Cycle** | | | |
| Isocitrate Lyase/malate synthase | C05E4.9 | GCTGTCAGTCGTGCGGTTAC | GCGGTGAGCGAAAGGATTT |
| **Anaerobic Pathways** | | | |
| Malic enzyme | Y48B6A.12 | CCAGCATCCGCCGTCTC | TTCCGGGCGGTAGAGCTT |
| **Hypoxia-inducible factor (HIF-1) pathway** | | | |
|  | *nhr-57* | GACTCTGTGTGGAGTGATGGAGAG | GTGGCTCTTGGTGTCAATTTCGGG |
|  | F22B5.4 | GAGATCCACGTTTTGTTAAAGTCGC | CGGCGGACAAGGAATTGATAAGGAG |
| **mtDNA copy number** | | | |
|  | nd1 | AGCGTCATTTATTGGGAAGAAGAC | AAGCTTGTGCTAATCCCATAAATGT |
| **Reference gene** |  |  |  |
| actin | *act-3* | TGCGACATTGATATCCGTAAGG | GGTGGTTCCTCCGGAAAGAA |
